# Dr. Herbert N. Tanner

**Published:** 1908-03

**Authors:** 


Dr. Herbert N. Tanner, formerly of Buffalo, more lately of East Aurora, N. Y., died at the Buffalo General Hospital, January 25, 1908, after a surgical operation, aged 38 years. Dr. Tanner was a graduate of Albany Medical College, 1895.

